# Association between Parkinson's disease and the risk of adverse cardiovascular events: a systematic review and meta-analysis

**DOI:** 10.3389/fcvm.2023.1284826

**Published:** 2023-12-07

**Authors:** Yan Hu, Shanxia Xu

**Affiliations:** Department of Neurology, Huzhou Central Hospital, Affiliated Central Hospital of HuZhou University, Huzhou, Zhejiang, China

**Keywords:** neurodegenerative disease, cardiac disease, mortality, risk, stroke

## Abstract

**Background:**

This review aims to examine the association of Parkinson's disease (PD) with the increased risk of cardiovascular events.

**Methods:**

PubMed, Embase, CENTRAL, and Scopus databases were electronically searched for papers published up to 5 May 2023. Studies reporting the association between PD and the subsequent risks of stroke, myocardial infarction (MI), and cardiovascular mortality were included.

**Results:**

Sixteen studies were included in this review. The clinical data of 101,712 PD patients were compared with that of the control group of 204,901 patients without PD in the included studies. Meta-analysis showed that PD patients had an increased risk of stroke compared with patients without PD (odds ratio (OR): 1.49; 95% confidence interval (CI): 1.30, 1.72; *I*^2 ^= 76%). The pooled analysis demonstrated no significant increase in the risk of MI (OR: 1.16; 95% CI: 0.85, 1.59; *I*^2 ^= 82%) and cardiovascular mortality (OR: 1.20; 95% CI: 0.93, 1.54; *I*^2 ^= 65%) in PD patients. However, data from cohort studies indicated a possibility of higher risk of MI (OR: 1.36; 95% CI: 1.01, 1.84; *I*^2 ^= 80%) and cardiovascular mortality (OR: 1.22; 95% CI: 1.00, 1.60; *I*^2 ^= 62%) in patients with PD.

**Conclusion:**

Patients with PD may have an increased risk of stroke as compared with the age- and gender-matched general population. While our results show that PD does not increase the overall risk of MI and cardiovascular mortality, analysis of cohort studies alone demonstrated that these risks may be higher in patients with PD. The current evidence is of very low quality. Further prospective cohort studies from different countries that would account for important cardiovascular risk factors are needed to improve the current evidence.

**Systematic Review Registration:**

https://www.crd.york.ac.uk/prospero/, PROSPERO (CRD42023421924).

## Introduction

Parkinson's disease (PD) is a progressive neurodegenerative movement disorder that commonly affects old people. The European data show that the prevalence rate of PD is about 108–257 per 100,000 individuals with an annual incidence of around 11–19 per 100,000 people (1). Data from the Chinese population show that around 1.37% of the aging population is affected by PD, that is, a total of 3.62 million patients (2). PD leads to movement abnormalities and other non-motor symptoms like memory loss, depression, visual disturbances, and autonomic dysfunction. The significant disability and poor quality of life of patients with PD are associated with a high economic burden on the healthcare system, and with increased physical and emotional dependence of patients on caregivers (3). These patients usually require full-time nursing care for their day-to-day activities and daily monitoring of the illness.

The aging population is also at an increased risk of cardiovascular diseases, a common cause of mortality in this age group. According to the American Heart Association on Heart Disease and Stroke Statistics (4), the risk of cardiovascular diseases rises from 35% to 40% in the 40–60 years age group to 75%–78% in the 60–80 years age group and exceeds 85% in people over 80 years of age. The accumulation of cardiovascular risk factors such as diabetes and obesity over a period of time also has a deleterious effect on the overall health of the aging population (5).

Currently, the association of PD with a higher risk of important cardiovascular events like stroke and myocardial infarction (MI) is not clear. Recently, a systematic review by Alves et al. (6) attempted to answer this clinical question, but it could only include 11 studies. The present updated meta-analysis aims to assess if PD increases the risk of cardiovascular events as compared with the age- and gender-matched control group.

## Material and methods

### Search source and strategy

The protocol of the review was prepared and published on PROSPERO (CRD42023421924) before beginning the study. A systematic search of PubMed, Embase, CENTRAL, and Scopus databases was carried out independently by two reviewers. The last date of the search was 5 May 2023. In addition, gray literature was searched by means of Google Scholar.

We aimed to include the following studies: (1) Cohort or case-control in design. (2) Studies comparing patient’s with PD with the general population matched at least for age and sex. (3) Studies examining the risk of stroke, MI, or cardiovascular mortality. (4) Studies reporting the effect size of the association or providing sufficient data to calculate the same.

The exclusion criteria were as follows: (1) Studies not on any cardiovascular outcome. (2) Cross-sectional studies. (3) Abstracts, editorials, thesis, and non-peer-reviewed studies. (4) In cases of duplication of data source, only a study with a higher sample size or better matching of cases and controls was included.

To conduct a wide but targeted search, we employed the following keywords: *Parkinson's disease*; *cardiac*; *cardiovascular*; *heart failure*; *myocardial infraction*; *coronary artery disease*; *cerebrovascular*; *stroke*; *mortality*; and *cardiovascular mortality*. Further details are mentioned in Supplementary Material Table S1.

### Study selection

Two reviewers independently examined all the search results. First, the retrieved data were congregated and deduplicated electronically. The titles and abstracts of all articles were screened to identify relevant studies. Non-relevant articles were excluded, and the remaining studies underwent full-text analysis based on the eligibility criteria. Any disagreements were solved by consensus. We also examined the reference lists of the included studies for any other missed articles.

### Extracted data and outcomes

Two reviewers were independently involved in the data extraction. A preformatted table was generated before beginning the review. The extracted data included the name(s) of the author(s), year of publication, location and database used, study type, sample size (PD and control), age and gender of the sample, diagnosis of PD, disease duration, matched variables, outcome reported, measurement of outcome, confounders adjusted, and follow-up period. Study details were then cross-matched, and any discrepancies were resolved by consensus.

The outcome of the review was selected based on the data reported by the studies. Quantitative analysis was done if at least four studies reported the same variable. Based on the initial analysis, stroke, MI, and cardiovascular mortality were selected as the outcomes of this review.

### Risk of bias analysis

Two reviewers assessed the quality of the included studies using the ROBINS-1 tool (7). The tool has seven domains of bias. Each one of the domains is examined using a series of signaling questions that extract information about the study and the analysis being done. Once the signaling questions are completed, the results are classified as serious risk of bias, moderate risk of bias, or low risk of bias. The Grading of Recommendations Assessment, Development, and Evaluation (GRADE) approach was also utilized using the GRADEpro GDT software for examining the certainty of the evidence.

### Statistical analysis

The review was conducted as per the Preferred Reporting Items for Systematic Reviews and Meta-Analyses (PRISMA) guidelines (8). The quantitative analysis was carried out using the software ReviewManager [RevMan, version 5.3; Nordic Cochrane Centre (Cochrane Collaboration), Copenhagen, Denmark; 2014]. The effect size of the association between PD and cardiovascular outcomes was extracted and combined using the generic inverse variation function of RevMan to generate an odds ratio (OR) with 95% confidence intervals (CI). Results were presented in the form of a forest plot. The meta-analysis was conducted in a random-effects model. And the subgroup analysis was done based on study type.

Funnel plots were generated using RevMan to judge publication bias. Interstudy heterogeneity was assessed by *I*^2^ statistics. A value of *I*^2^ < 50% indicated low heterogeneity and that >50% indicated substantial heterogeneity. A sensitivity analysis was performed to check for outliers in the analysis. One study at a time was removed from the meta-analysis and the results were regenerated.

## Results

### Search results

**Figure 1:**
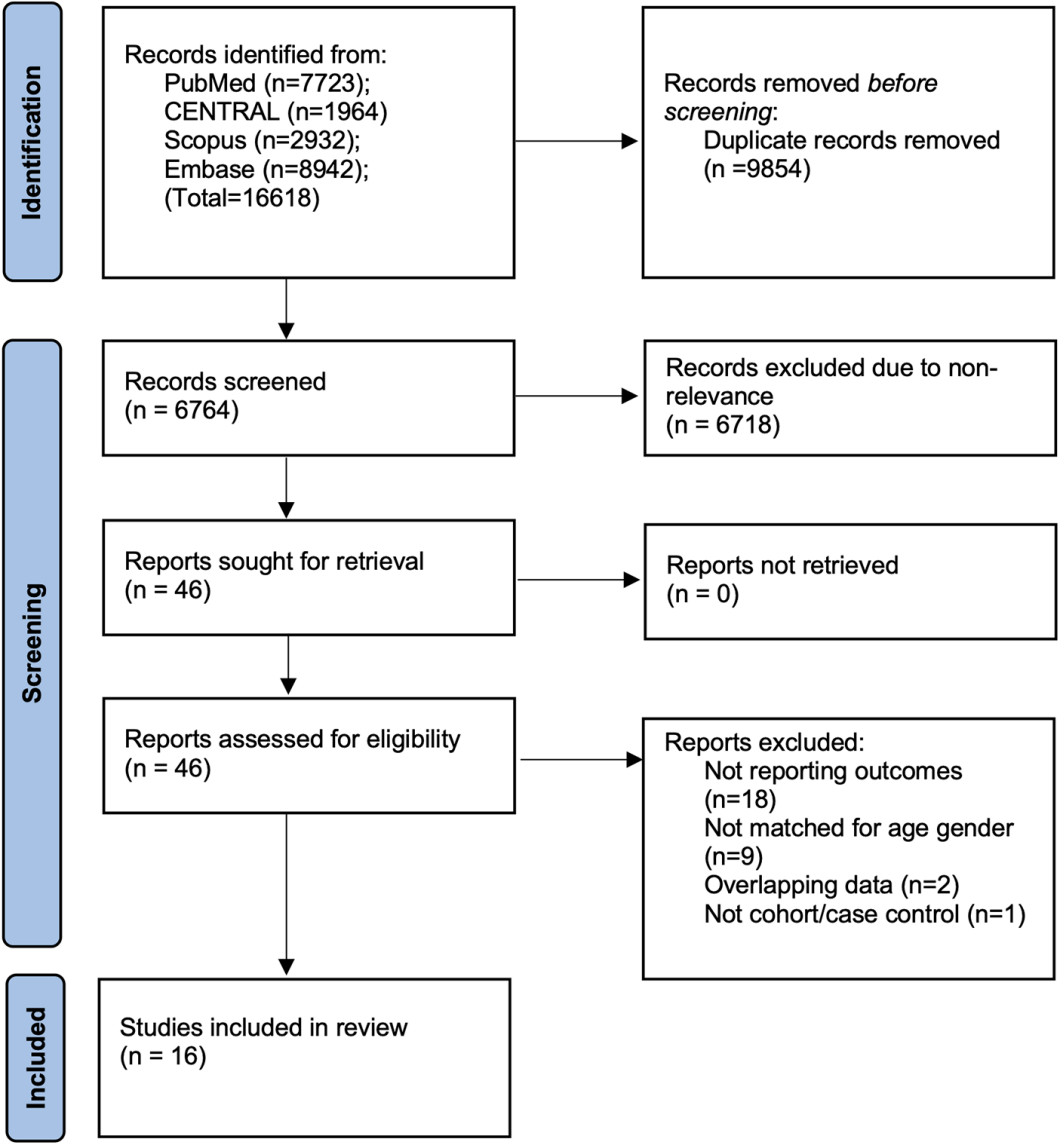
Study flowchart.

The results generated during the search strategy are shown in Figure 1. A total of 16,618 articles were initially retrieved. Of them, 6,764 records remained after deduplication, and 46 were subsequently found suitable for full-text analysis. Eventually, 16 studies were included in the final review (9–24). The inter-reviewer agreement for the selection of studies was high (kappa = 0.95). No additional studies were found on Google Scholar.

### Study details

The data sourced from the included studies are provided in Table 1. These studies were published over a wide timeline, ranging from 1992 to 2022. The study locations were the USA, the UK, Sweden, Ireland, Italy, Spain, South Korea, and Taiwan. All studies were retrospective. Four were case-control studies, while the remaining 12 were cohort studies. A total of 101,712 patients with PD were compared with a control group of 204,901 individuals without PD in the included studies. PD was diagnosed clinically in only four studies, while the rest used medical records to identify patients with PD. The study and control groups in the included studies were matched for age, gender, and other different variables. Most studies used medical records to identify the outcome of interest. There was a high variability in the follow-up duration in the included studies. The risk of bias assessment is presented in Table 2. Five studies had a low risk of bias, two had a moderate risk of bias, while the remaining had a high risk of bias.

**Table 1:** Details of the included studies

Study, year	Location	Data source	Type	Sample size		Age (years)	Male gender (%)	Diagnose of PD	Disease duration (years)	Matched variables	Outcome	Outcome assessment	Confounders adjusted	Follow-up (years)
				PD	Control									
Levine (24)	USA	Medical record of discharged patients	C	119	238	68.5	100	Neurologist or review of medical records	7.3	Age, sex	MI, stroke	Medical records	NR	8
Ben-Shlomo (23)	Ireland	Records from the Second National Morbidity study	C	195	295	69	40.6	ICD codes	NR	Practice, age, sex	MI, CVM	ICD codes	Age, sex, and region	20
Fall (22)	Sweden	Epidemiology study	C	170	510	73.7	NR	Examination of patients and medical records by neurologist	12.8 ± 8.5	Postal area, age, sex	MI, stroke, CVM	ICD codes	NR	9
D’Amelio (21)	Italy	The Sicilian neuroepidemiologic study	C	58	116	75	44.1	Screening questionnaire and neurologist evaluation	5.5	Age, sex, municipally of residence	Stroke, CVM	Death certificate	NR	7–10
Driver (17)	USA	Physician's health study	C	560	560	73.3	100	Self-reported	2–10	Age, sex, Charlson comorbidity index	MI, stroke, CVM	ICD codes	Age at onset and smoking status	24
Becker (18)	UK	General practice research database	C	298	321	NR	59.6	Oxford medical information system	≥3	Age, sex, calendar time	Stroke	N/R	BMI, smoking, arrhythmias, ischemic heart disease, hypertension, diabetes, dementia, depression, use of anti-depressants	21
Patel (19)	UK	Royal Preston Hospital	CC	85	85	67	65	Clinical diagnosis	6.5	Age, gender, and modality of cranial imaging	Stroke, MI	Medical records	None	NR
Huang (20)	Taiwan	National Health Insurance database	C	2,204	2,204	71	49.6	ICD codes	<3	Age, sex	Stroke	ICD codes	Propensity score matched	3
Braga (16)	Italy	Vimercate hospital	CC	285	285	NR	50	ICD codes	NR	Ethnicity, sex and age, and hospitalized in the same year	Stroke, MI	Medical records	None	NR
Liang (14)	Taiwan	National Health Insurance database	C	3,211	3,211	71	49.6	ICD codes	<3	Age, sex	MI, CVM	ICD codes	Propensity score matched	3
Hobson (15)	UK	Medical records of general practitioners	C	158	34	74.2	56	Medical records	13.2	Age, sex	MI, stroke, CVM	ICD codes	NR	2
Choi (13)	South Korea	Korean National Health Insurance Service	C	3,510	14,040	NR	38.1	ICD codes	NR	Age group, sex, income, region of residence and medical histories of hypertension, diabetes mellitus, and dyslipidaemia	CVM	ICD codes	Age, sex, income, and region of residence	4
Abugroun (12)	USA	National inpatient sample	CC	54,882	54,882	79	57.5	ICD codes	NR	Age, sex	Stroke	ICD codes	Propensity score matched	NR
Park (10)	South Korea	Korean National Health Insurance Service	C	25,624	128,120	NR	NR	ICD codes	NR	Age, sex	MI, stroke	ICD codes	Age, sex, household income, hypertension, diabetes mellitus, dyslipidemia, chronic obstructive pulmonary disease, and end-stage renal disease	1–6
Macías-García (11)	Spain	Hospital Universitario Virgen del Rocío	CC	355	620	NR	NR	Clinical diagnosis	8–22	Age, sex, and vascular risk factors	Stroke	Clinical diagnosis	Age, sex, and vascular risk factors	NR
Okunoye (9)	UK	IQVIA medical research data	C	9,998	55,554	NR	60.7	Read codes and prescription of medications	NR	Age, gender, calendar year, and practice	Stoke, MI	Read codes	Age, gender, calendar year, social deprivation, and smoking	5

C, cohort; CC, case control; ICD, international classification of diseases; NR, not reported; PD, Parkinson's disease; MI, myocardial infarction; CVM, cardiovascular mortality; NOS, Newcastle Ottawa scale.

**Table 2:** Risk of bias analysis using the ROBINS-1 tool

Study, year	Bias due to confounding	Bias in selection of participants	Bias in classification of intervention	Bias due to deviation from intended intervention	Bias due to missing data	Bias in measurement of outcomes	Bias in selection of reported results	Overall risk of bias
Levine (24)	Serious	Low	Low	Low	Low	Low	Low	Serious
Ben-Shlomo (23)	Serious	Low	Low	Low	Low	Low	Low	Serious
Fall (22)	Serious	Low	Low	Low	Low	Low	Low	Serious
D’Amelio (21)	Serious	Low	Low	Low	Low	Low	Low	Serious
Driver (17)	Serious	Low	Serious	Low	Low	Low	Low	Serious
Becker (18)	Low	Low	Low	Low	Low	Low	Low	Low
Patel (19)	Serious	Low	Low	Low	Low	Low	Low	Serious
Huang (20)	Low	Low	Low	Low	Low	Low	Low	Low
Braga (16)	Serious	Low	Low	Low	Low	Low	Low	Serious
Liang (14)	Low	Low	Low	Low	Low	Low	Low	Low
Hobson (15)	Serious	Low	Low	Low	Low	Low	Low	Serious
Choi (13)	Low	Low	Low	Low	Low	Low	Low	Low
Abugroun (12)	Moderate	Low	Low	Low	Low	Low	Low	Moderate
Park (10)	Low	Low	Low	Low	Low	Low	Low	Low
Macías-García (11)	Moderate	Low	Low	Low	Low	Low	Low	Moderate
Okunoye (9)	Moderate	Low	Moderate	Low	Low	Low	Low	Serious

### Outcomes

**Figure 2:**
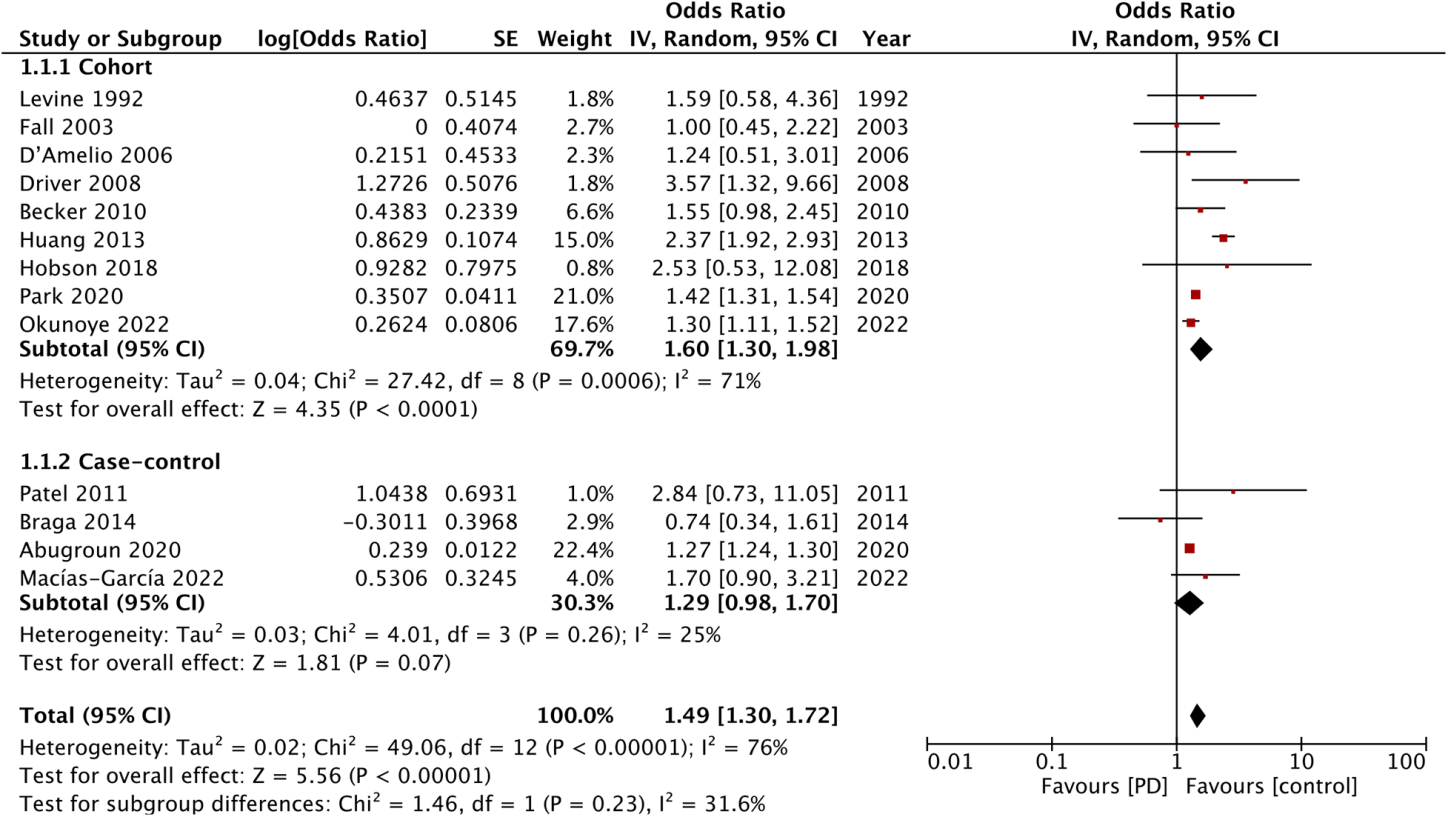
Meta-analysis of the risk of stroke among patients with PD and non-PD controls.

A total of 13 studies reported data on stroke. The meta-analysis showed that patients with PD had an increased risk of stroke as compared with the non-PD group (OR: 1.49; 95% CI: 1.30, 1.72; *I*^2 ^= 76%) (Figure 2). The results were significant for the cohort studies (OR: 1.60; 95% CI: 1.30, 1.98; *I*^2 ^= 71%) but not for the case-control studies (OR: 1.29; 95% CI: 0.98, 1.70; *I*^2 ^= 25%). Visual inspection of the funnel plot did not show any publication bias (Supplementary Material Figure S1). Furthermore, sensitivity analysis did not detect any effect of the singular exclusion of individual studies.

**Figure 3:**
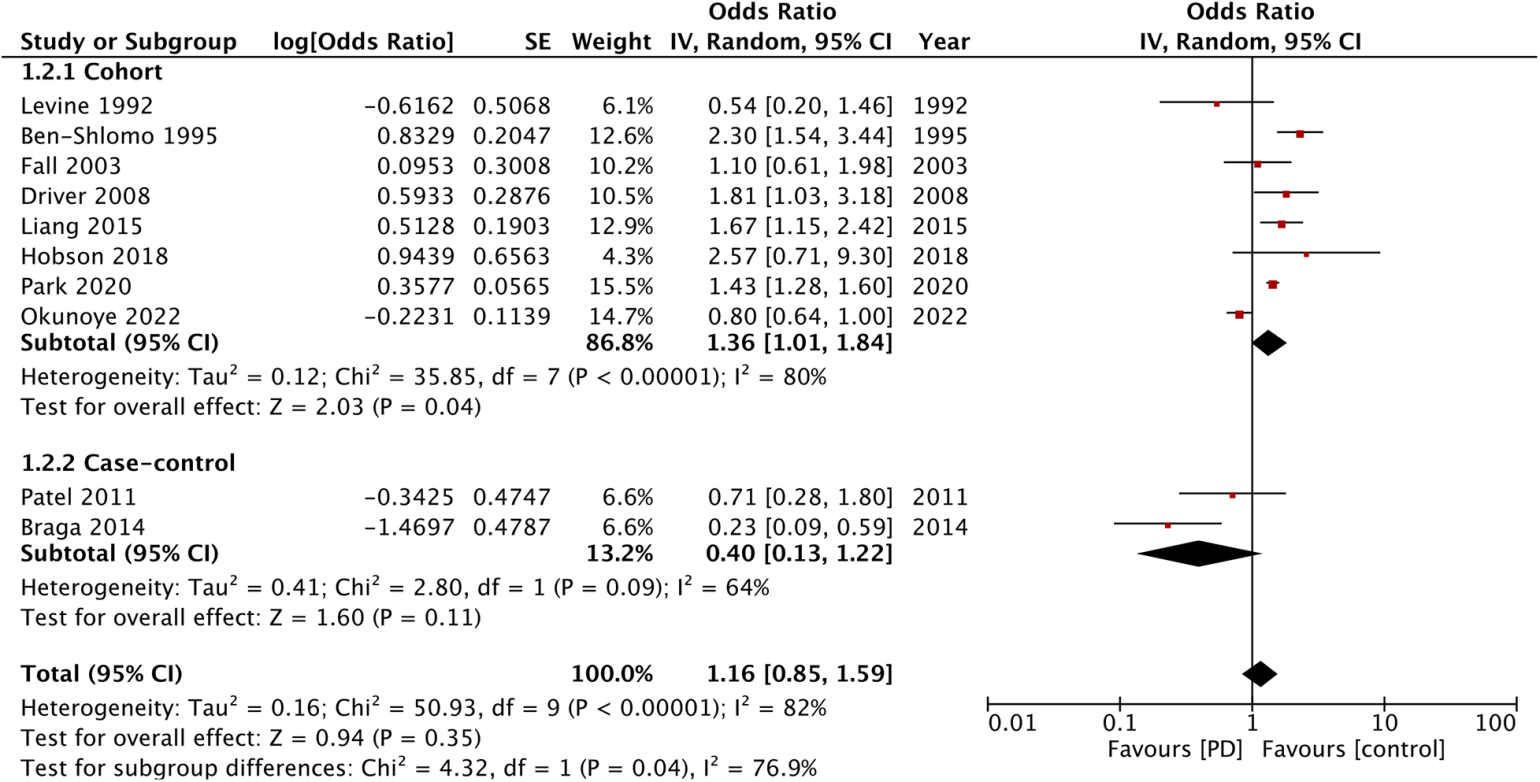
Meta-analysis of the risk of MI among patients with PD and non-PD controls.

For the outcome of MI, 10 studies were available. A pooled analysis demonstrated no significant difference in the risk of MI in patients with PD as compared with the non-PD group (OR: 1.16; 95% CI: 0.85, 1.59; *I*^2 ^= 82%) (Figure 3). However, the results of the cohort studies (OR: 1.36; 95% CI: 1.01, 1.84; *I*^2 ^= 80%) showed a significant increase in the risk of MI in patients with PD, while no such association was noted for the case-control studies (OR: 0.40; 95% CI: 0.13, 1.22; *I*^2 ^= 64%). Visual inspection of the funnel plot did not show any publication bias (Supplementary Material Figure S2), and sensitivity analysis did not detect any change in the results.

**Figure 4:**
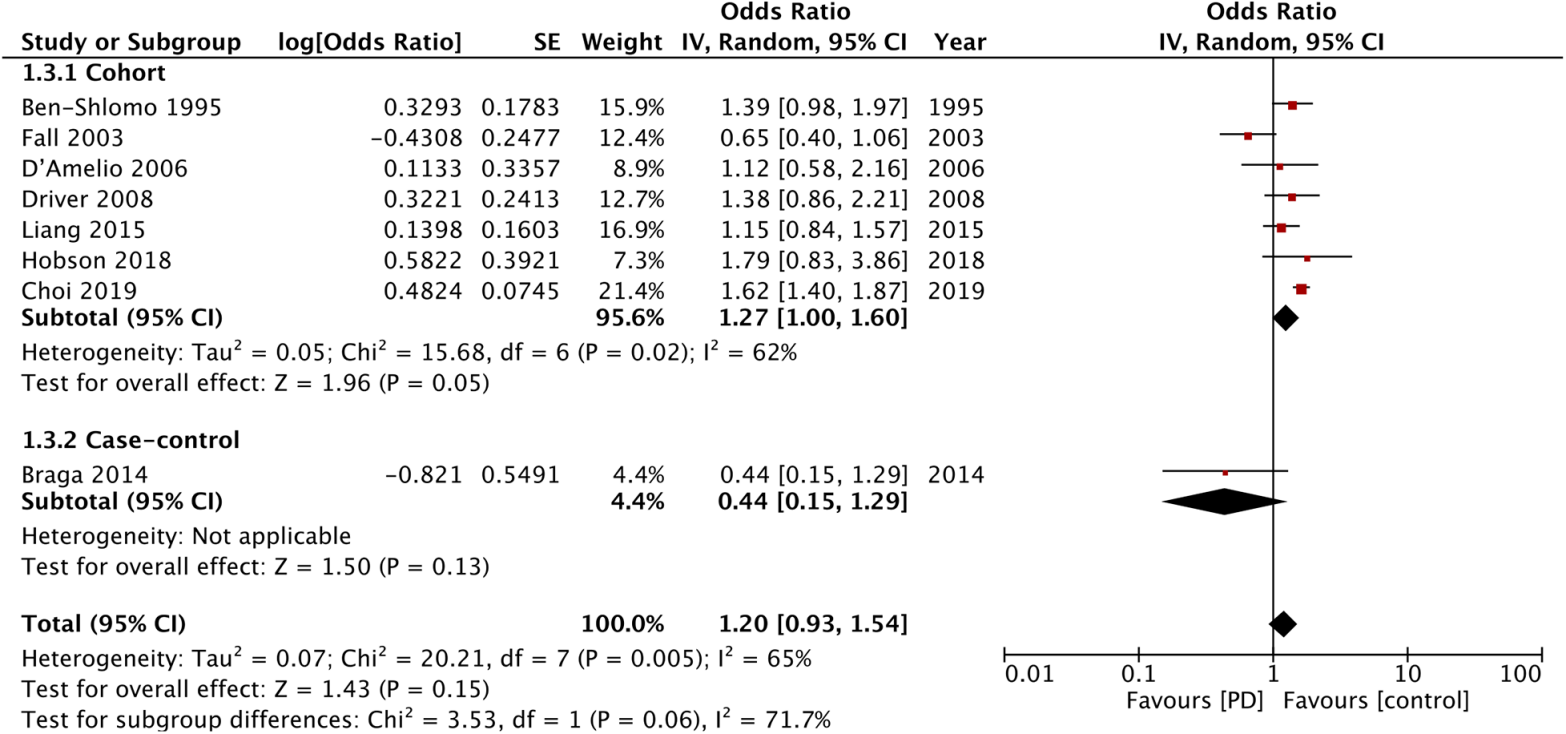
Meta-analysis of the risk of cardiovascular mortality among patients with PD and non-PD controls.

Eight studies reported data on cardiovascular mortality. The meta-analysis found no difference in cardiovascular mortality between the PD and non-PD groups (OR: 1.20; 95% CI: 0.93, 1.54; *I*^2 ^= 65%). Sensitivity analysis showed that the exclusion of the study of Fall et al. (14) resulted in a significant increase in the risk of cardiovascular mortality (OR: 1.36; 95% CI: 1.11, 1.65; *I*^2 ^= 40%). There was a tendency for increased cardiovascular mortality in the cohort studies (OR: 1.22; 95% CI: 1.00, 1.60; *I*^2 ^= 62%) but not for the case-control studies (OR: 0.44; 95% CI: 0.15, 1.29) (Figure 4).

### Certainty of evidence

GRADE assessment of evidence is provided in the Supplementary Material Table S2. Since the majority of the studies had serious bias in the adjustment of confounders, the certainty of evidence was downgraded. Overall, all outcomes had *very low* certainty of evidence.

## Discussion

This updated systematic review and meta-analysis included 16 studies examining the association between PD and adverse cardiovascular outcomes. Our results showed that PD was associated with a 49% higher risk of stroke as compared with the age- and gender-matched general population. Nevertheless, there was no statistically significant association between PD and the risk of MI or cardiovascular mortality. Our results concur with the previous review (6) on this topic, but with a higher number of studies.

With a gradual aging of the global population, the prevalence of age-related neurological and cardiovascular disorders is also on the rise (25). PD constitutes one of the major neurological disorders that causes significant disability and results in a poor quality of life in the aging population (1). Importantly, this group is also at a higher risk of cardiovascular disorders. Comorbidities like stroke and MI could worsen the prognosis of PD. Therefore, understanding the potential impact of PD on cardiovascular outcomes in aging patients is crucial. This review examined the association between PD and three important cardiovascular outcomes, namely, stroke, MI, and cardiovascular mortality. It is important to note that age and gender could be important confounders while assessing such associations. If the control group is younger, the risk could be overestimated leading to false positive results. Therefore, we included only studies with age- and gender-matched controls to generate better-quality evidence.

The pooled analysis of 13 studies found a significantly higher risk of stroke in patients with PD. When stratified based on the study type, the association was significant for the cohort but not for the case-control studies. The cohort studies are a better source of scientific evidence as they have a temporal framework to assess causality (26). Also, the number of cohort studies was higher compared with the case-control studies in our analysis. The lack of change in the significance of the results, demonstrated by the sensitivity analysis, also adds to the credibility of this outcome.

There are several mechanisms by which PD could increase the risk of stroke (27). Motor dysfunction leading to reduced physical activity is the hallmark of PD. Several studies have shown that physical activity is important for stroke prevention (28, 29). Second, autonomic dysfunction, which affects >40% of patients with PD (30), is an important regulator of the heart–brain axis and could be a key factor in the heightened risk of stroke. According to one study (30), about a third of the patients with PD have orthostatic hypotension owing to the autonomic dysfunction. Another study with almost 25 years of follow-up has demonstrated a twofold higher risk of stroke in patients with orthostatic hypotension (31). It is plausible that the episodes of hypotension could lead to brain hypoperfusion, thereby increasing the risk of ischemic stroke.

Another possible mechanism for the association between PD and stroke may be related to homocysteine metabolism. Homocysteine is an amino acid that has a proinflammatory effect on arteries and is a recognized risk factor for cardiovascular and neurodegenerative disease (32). Studies show that patients with PD may have increased levels of homocysteine compared with controls, and the difference is more pronounced in patients who were treated with L-dopa (33, 34). Homocysteine with its proinflammatory function has been shown to increase oxidative stress and reduce nitric oxide synthesis, thereby causing endothelial dysfunction (35). Clinically, it has been associated with atherosclerosis and an increased risk of stroke (36).

Stroke and coronary artery disease have several common risk factors. However, while PD in our study was associated with the increased risk of stroke, this meta-analysis did not find such a correlation between the risk of MI and cardiovascular mortality. The analysis of only the cohort studies did demonstrate a 36% increased risk of MI and a 22% increased risk of cardiovascular mortality. In addition, we identified one outlier study (14) in the analysis of cardiovascular mortality. Its exclusion resulted in a 36% increased risk of cardiovascular mortality, associated with PD. The methodological differences in the study designs could have been important factors leading to such variability in the results. More importantly, the number of cohort studies in the meta-analysis of MI and cardiovascular mortality was not high, and the high interstudy heterogeneity prohibits wider interpretation of the results.

A recent review of cohort, case-control, and cross-sectional studies has also demonstrated no association between PD and MI (37). Another review by Chua et al. (38), published in 2022, combined data from three cohort studies and two case-control studies examining the relationship between PD and coronary artery disease. Similarly to our study, this review showed a significant association between PD and coronary artery disease in cohort studies but not in case-control studies. Hence, it seems that the design of the study is an important component in assessing the relationship between PD and cardiovascular events.

Mechanisms that increase the risk of stroke have also been linked with the pathogenesis of MI in patients with PD. A meta-analysis of 13 studies has shown that orthostatic hypotension is associated with an increased risk of coronary artery disease, heart failure, and all-cause mortality (39). Patients with PD suffer from baroreflex dysfunction, which increases sympathetic activity and reduces parasympathetic control, leading to reduced diastolic perfusion pressure and reduced myocardial blood flow (40). Orthostatic hypotension also activates compensatory neuroendocrine mechanisms, which in turn could trigger platelets and coagulation factors and lead to adverse cardiovascular events (39). One such example includes changes in the endothelin system that are seen in cases of orthostatic hypotension. Physiological vasoconstrictors like endothelin-1 and vasopressin that have an adaptive role in hypotensive episodes could increase the risk of atherothrombosis in the long term (41).

Second, the higher levels of homocysteine in patients with PD can lead to a hypercoagulable state that increases the risk of coronary thrombosis and adverse cardiovascular events (34–36). In addition, inflammation could be a shared risk factor between PD and MI. Chronic neuroinflammation causing activation of microglia and elevated levels of proinflammatory mediators has been an important contributor to the pathophysiology of PD (42). Correspondingly, oxidative stress and systemic inflammation are also known risk factors for atherosclerosis and coronary artery disease (43). Therefore, it is plausible that the baseline systemic inflammation is higher in patients with PD and contributes to the increased risk of MI.

There are several limitations to this review. The number of studies in each analysis was not very high, especially in terms of cardiac outcomes. The high heterogeneity is a significant limitation and could be due to variations in study populations, methodology, and region of the study. Furthermore, there were differences in the methods used to diagnose patients with PD. Only four studies performed a clinical diagnosis, while the rest relied on medical records, which could introduce an important source of bias. Also, there was no uniformity in the adjusted confounders among the included studies. Important cardiovascular risk factors, such as diabetes, hypertension, obesity, and smoking, were not adjusted in multiple studies, which can potentially skew the results. In addition, we observed variability in the duration of follow-ups. Currently, it is unclear how long it takes for PD to increase the risk of adverse cardiovascular events. Therefore, studies with shorter follow-ups could potentially miss such outcomes. Finally, owing to the unavailability of data, other important cardiovascular outcomes like heart failure could not be examined by this review.

The strength of this review lies in its updated and comprehensive literature search involving several databases. Importantly, only age- and gender-matched studies were included to optimize the outcomes. Also, we avoided cross-sectional studies due to their inherent bias and inability to demonstrate causality (26). Separate analysis for the case-control and the cohort studies was conducted with sensitivity analysis to provide a detailed evaluation of the results.

The results have clinical implications. Patients with PD should be considered at high risk for adverse cardiovascular events. They should be closely monitored, especially by nursing personnel involved in their care, for acute symptoms of stroke, MI, and other cardiovascular diseases. However, it must also be considered that the quality of evidence in our study was low. The certainty of evidence was downgraded due to the risk of bias in the included studies as a result of the lack of adjustment of confounders. Therefore, our results should be interpreted with caution, and there is a need to strengthen the evidence by conducting more robust studies. Studies should also assess the pathophysiological relationship between PD and cardiovascular events and the possible interventional strategies to reduce such risk.

## Conclusions

Patients with PD may have an increased risk of stroke as compared with the age- and gender-matched general population. Overall, our results show that PD does not increase the risk of MI and cardiovascular mortality. However, analysis of only the cohort studies demonstrated that patients with PD may have an increased risk of MI and cardiovascular mortality. Current evidence is of very low quality. Further prospective cohort studies from different countries and taking into account important cardiovascular risk factors are needed to improve the current evidence.

## Data Availability

The original contributions presented in the study are included in the article/**Supplementary Materials**, further inquiries can be directed to the corresponding author.
