# Supplementary material for: Association between Parkinson's disease and the risk of adverse cardiovascular events: a systematic review and meta-analysis
Source: Front Cardiovasc Med. 2023 Dec 7;10:1284826. doi: 10.3389/fcvm.2023.1284826 (PMC10748497; doi:10.3389/fcvm.2023.1284826)
Supplement: Supplementary file 1 [file Table1.docx]

Supplementary Table 1: Search strategy

| **Query** |
| --- |
| ((mortality[Title/Abstract]) OR (cardiovascular mortality[Title/Abstract])) AND (Parkinson’s disease[Title/Abstract]) |
| ((cerebrovascular[Title/Abstract]) OR (stroke[Title/Abstract])) AND (Parkinson’s disease[Title/Abstract]) |
| (((heart failure[Title/Abstract]) OR (myocardial infarction[Title/Abstract])) OR (coronary artery disease[Title/Abstract])) AND (Parkinson's disease[Title/Abstract]) |
| ((cardiac[Title/Abstract]) OR (cardiovascular mortality[Title/Abstract])) AND (Parkinson’s disease[Title/Abstract]) |
