# Supplementary material for: Association between Parkinson's disease and the risk of adverse cardiovascular events: a systematic review and meta-analysis
Source: Front Cardiovasc Med. 2023 Dec 7;10:1284826. doi: 10.3389/fcvm.2023.1284826 (PMC10748497; doi:10.3389/fcvm.2023.1284826)
Supplement: Supplementary file 2 [file Table2.docx]

**Supplementary Table 2: GRADE assessment of evidence**

| **Certainty assessment** | | | | | | | **Effect** | | **Certainty** | **Importance** |  |
| --- | --- | --- | --- | --- | --- | --- | --- | --- | --- | --- | --- |
| **№ of studies** | **Study design** | **Risk of bias** | **Inconsistency** | **Indirectness** | **Imprecision** | **Other considerations** | **Relative (95% CI)** | **Absolute (95% CI)** |  |  |  |
| **Stroke - Cohort** | | | | | | | | | | | |
| 9 | observational studies | very serious^a^ | not serious | not serious | not serious | none | **OR 1.60** (1.30 to 1.98) | **2 fewer per 1,000** (from 2 fewer to 1 fewer) | ⨁◯◯◯ Very low |  |  |
| **Stroke - Case-control** | | | | | | | | | | | |
| 4 | observational studies | very serious^a^ | not serious | not serious | not serious | none | **OR 1.29** (0.98 to 1.70) | - | ⨁◯◯◯ Very low |  |  |
|  |  |  |  |  |  |  |  | **0 fewer per 1,000** (from 0 fewer to 0 fewer) |  |  |  |
| **MI - Cohort** | | | | | | | | | | | |
| 8 | observational studies | very serious^a^ | not serious | not serious | not serious | none | **OR 1.36** (1.01 to 1.84) | **1 fewer per 1,000** (from 2 fewer to 1 fewer) | ⨁◯◯◯ Very low |  |  |
| **MI - Case-control** | | | | | | | | | | | |
| 2 | observational studies | very serious^a^ | not serious | not serious | serious^b^ | none | **OR 0.40** (0.13 to 1.22) | - | ⨁◯◯◯ Very low |  |  |
|  |  |  |  |  |  |  |  | **0 fewer per 1,000** (from 0 fewer to 0 fewer) |  |  |  |
| **Cardiovascular mortality - Cohort** | | | | | | | | | | | |
| 6 | observational studies | very serious^a^ | not serious | not serious | not serious | none | **OR 1.49** (1.32 to 1.67) | **1 fewer per 1,000** (from 2 fewer to 1 fewer) | ⨁◯◯◯ Very low |  |  |

**CI:** confidence interval; **OR:** odds ratio

#### Explanations

a. Majority studies had serious or moderate risk of bias

b. very low number of studies
